# Supplementary material for: Swapped and non-swapped TRAAK states co-exist in membranes at a ratio influenced by temperature
Source: Nat Commun. 2026 Mar 5;17:3522. doi: 10.1038/s41467-026-70027-9 (PMC13087257; doi:10.1038/s41467-026-70027-9)
Supplement: Supplementary file 1 — Supplementary Information [file 41467_2026_70027_MOESM1_ESM.pdf]

Supplementary Information for

Swapped and non-swapped TRAAK states co-exist in membranes at a ratio influenced by temperature

Yue Ma[1,2], Katrin Ackermann[3], Qaiser Waheed[1,2], Vincent Postis[4], Terry K Smith[5], Bela E Bode[3] and Christos Pliotas[1,2]*

[1]BioEmPiRe Centre for Structural Biological EPR Spectroscopy, School of Biological Sciences, Faculty of Biology, Medicine and Health, The University of Manchester, Manchester, M13 9PT, United Kingdom
[2]Manchester Institute of Biotechnology, The University of Manchester, Manchester, M1 7DN, United Kingdom
[3]EaStCHEM School of Chemistry, Biomedical Sciences Research Complex and Centre of Magnetic Resonance, University of St Andrews, St Andrews, KY16 9ST, United Kingdom
[4]Wellcome Centre for Anti-Infectives Research, Division of Biological Chemistry and Drug Discovery, School of Life Sciences, University of Dundee, Dundee DD1 5EH, United Kingdom.
[5]School of Biology, Biomedical Sciences Research Complex, University of St Andrews, St Andrews, KY16 9ST, United Kingdom

*Corresponding author. Email: christos.pliotas@manchester.ac.uk

The file includes:

Supplementary Figures: 1 to 17
Supplementary Tables: 1 to 4

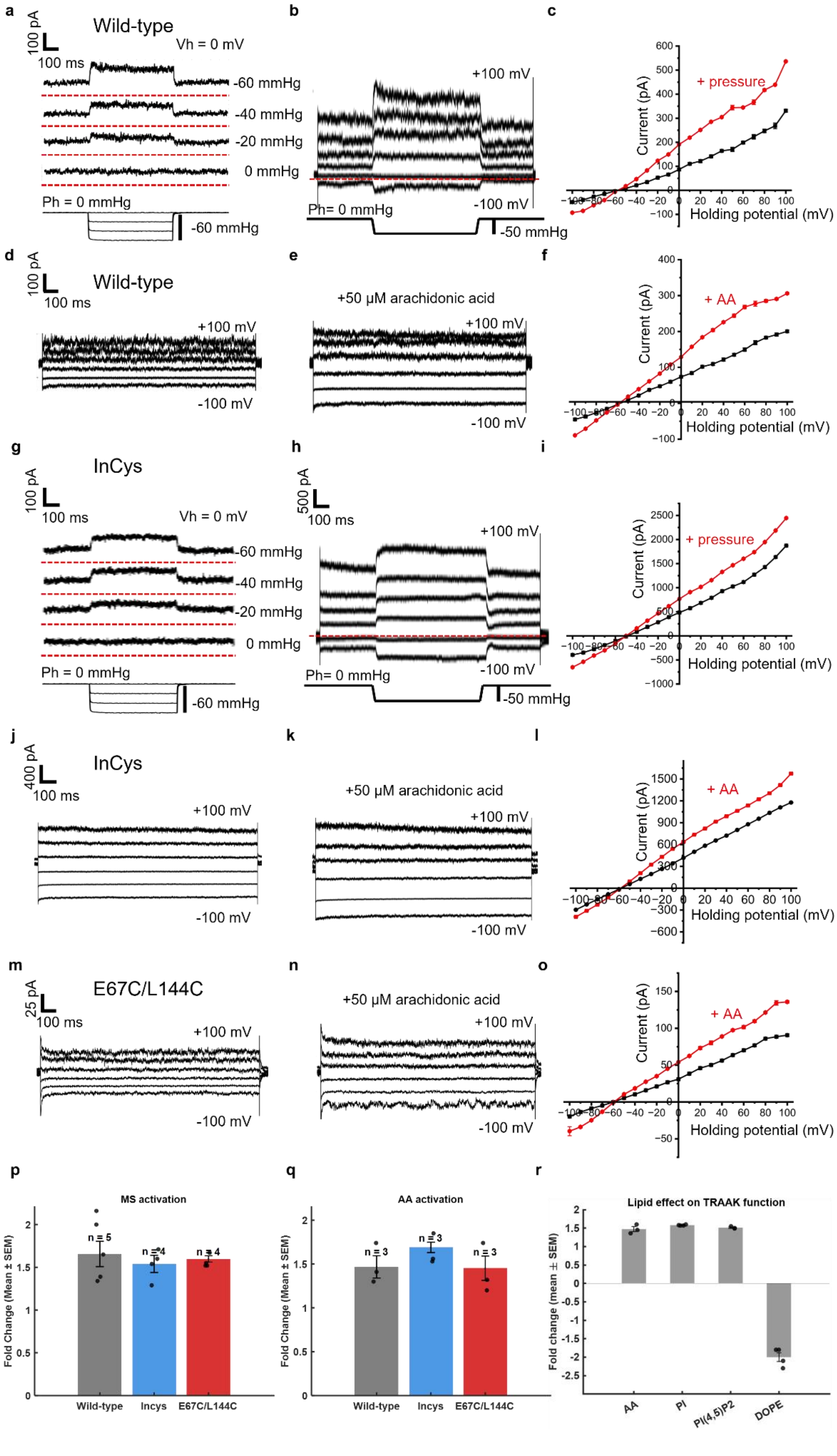
a
Wild-type
Vh = 0 mV
100 pA
100 ms
-60 mmHg
-40 mmHg
-20 mmHg
0 mmHg
Ph = 0 mmHg
-60 mmHg
b
+100 mV
-100 mV
Ph= 0 mmHg
-50 mmHg
c
+ pressure
Current (pA)
Holding potential (mV)
d
Wild-type
100 pA
100 ms
+100 mV
-100 mV
e
+50 μM arachidonic acid
+100 mV
-100 mV
f
+ AA
Current (pA)
Holding potential (mV)
g
InCys
100 pA
100 ms
Vh = 0 mV
-60 mmHg
-40 mmHg
-20 mmHg
0 mmHg
Ph = 0 mmHg
-60 mmHg
h
500 pA
100 ms
+100 mV
-100 mV
Ph= 0 mmHg
-50 mmHg
i
+ pressure
Current (pA)
Holding potential (mV)
j
InCys
400 pA
100 ms
+100 mV
-100 mV
k
+50 μM arachidonic acid
+100 mV
-100 mV
l
+ AA
Current (pA)
Holding potential (mV)
m
E67C/L144C
25 pA
100 ms
+100 mV
-100 mV
n
+50 μM arachidonic acid
+100 mV
-100 mV
o
+ AA
Current (pA)
Holding potential (mV)
p
MS activation
Fold Change (Mean ± SEM)
n = 5
n = 4
n = 4
Wild-type
Incys
E67C/L144C
q
AA activation
Fold Change (Mean ± SEM)
n = 3
n = 3
n = 3
Wild-type
Incys
E67C/L144C
r
Lipid effect on TRAAK function
Fold change (mean ± SEM)
AA
PI
PI(4,5)P2
DOPE

Supplementary Fig. 1: **Comparison of channel activity for wild-type, InCys, and E67C/L144C TRAAK.**

a, g, Current responses to incremental negative pressure applied to representative excised patches from wild-type (a) and InCys (g) TRAAK GUVs. Pressure steps every 5 s; traces vertically offset. Dashed red lines indicate the zero-current baseline. Holding potential (Vh) = 0 mV, holding pressure (Ph) = 0 mmHg. **b, h**, Currents recorded from patches of wild-type (b) and InCys (h) TRAAK during a voltage-step protocol (Vh=−50mV ; −100 to +100 mV in 10-mV increments; displayed every 40 mV). For each voltage step, a −50 mmHg pressure step was applied (lower). Dashed red line marks the current at Vh=−50 mV, Ph=0 mmHg. **c, I,** I–V relationships from (b, h): average pre-pressure current and peak current during the pressure step vs voltage. **d, j, m,** Currents from excised patches of wild-type (d), InCys (j), and E67C/L144C (m) under a voltage-step protocol (Vh=0 mV; −100 to +100 mV;ΔV=10 mV; displayed every 40 mV). **e, k, n**, Recordings from the same patches as (d, j, m) after 50 µM arachidonic acid (AA) perfusion. **f, l, o**, I–V relationships corresponding to (d/e), (j/k), and (m/n), respectively. **p, q,** Fold change in current at Vh = 0 mV for wild-type, InCys, and E67C/L144C TRAAK in response to MS (−50 mmHg) and AA stimulation. MS increased current 1.66 ± 0.15, 1.54 ± 0.10, and 1.60 ± 0.04-fold, respectively; AA increased current 1.47 ± 0.13, 1.69 ± 0.06, and 1.45 ± 0.14-fold, respectively (mean ± SEM). Biological replicate numbers (n) are shown above the bars. **r,** Fold change in current at Vh = 0 mV of wild-type TRAAK in response to lipid perfusion. TRAAK was activated 1.47 ± 0.13, 1.58 ± 0.02, and 1.51 ± 0.03-fold by AA, PI, and PI(4,5)P2, respectively (mean ± SEM, n = 3 patches). In contrast, DOPE inhibited TRAAK by 2.00 ± 0.25-fold (mean ± SEM, n = 3 patches).

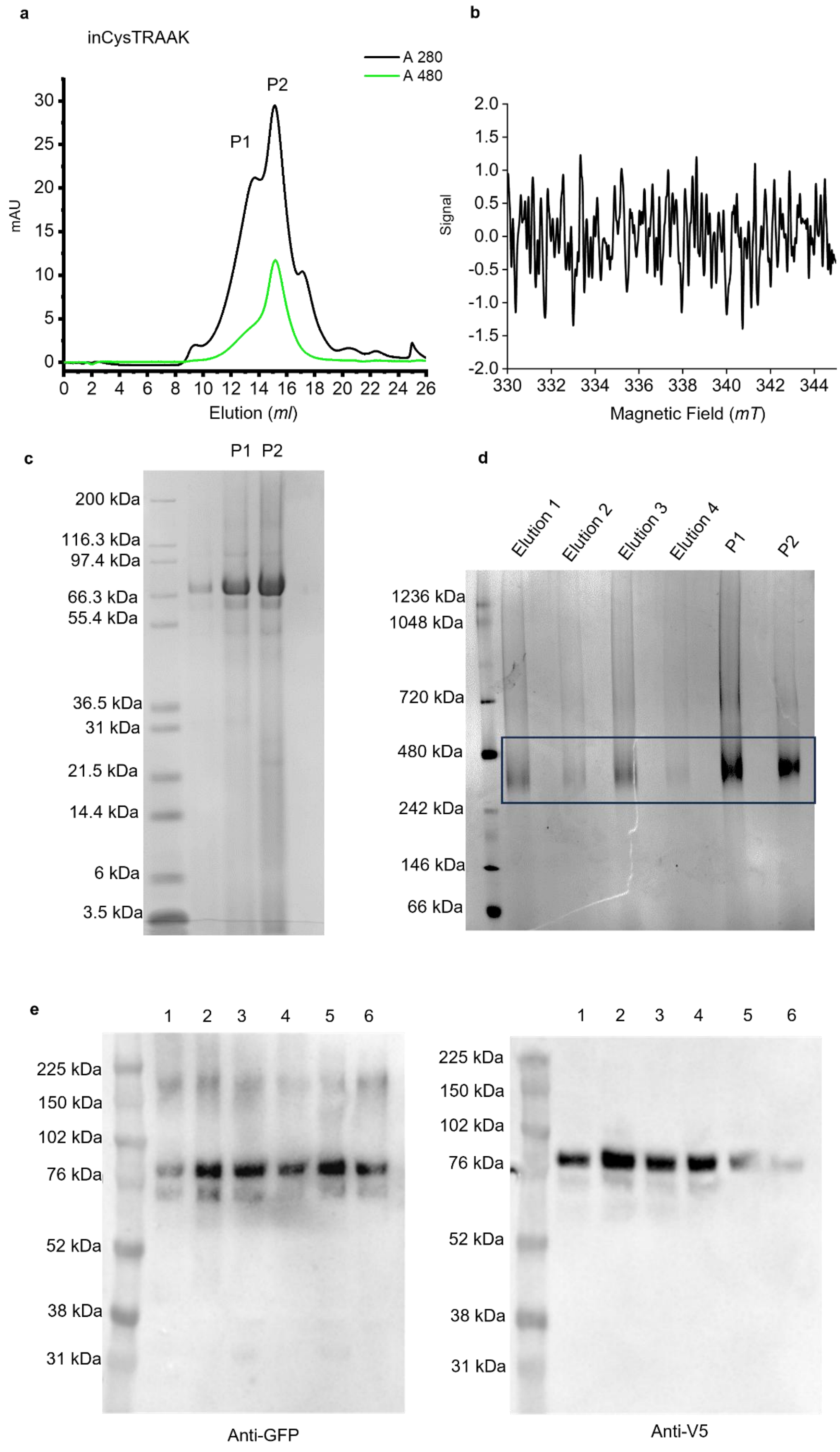
a
inCysTRAAK
A 280
A 480
P1
P2
mAU
Elution (ml)
b
Signal
Magnetic Field (mT)
c
P1 P2
200 kDa
116.3 kDa
97.4 kDa
66.3 kDa
55.4 kDa
36.5 kDa
31 kDa
21.5 kDa
14.4 kDa
6 kDa
3.5 kDa
d
Elution 1
Elution 2
Elution 3
Elution 4
P1
P2
1236 kDa
1048 kDa
720 kDa
480 kDa
242 kDa
146 kDa
66 kDa
e
1 2 3 4 5 6
225 kDa
150 kDa
102 kDa
76 kDa
52 kDa
38 kDa
31 kDa
Anti-GFP
Anti-V5

Supplementary Fig. 2: Biochemical analysis of full-length human TRAAK. **a**, Gel-filtration analysis of inCysTRAAK on Superose6 Increase column. Major fractions were labelled as P1 and P2. **b**, CW EPR spectrum of inCysTRAAK in SMALPs (fraction P2 in A) measured at 4°C. **c**, SDS-PAGE gel of fraction P1 and P2 from **a**. **d**, Blue Native (BN) Gel of 4 fractions following elution from a Ni^{2+}-NTA column and fractions from **a**. **e**, Western blot shows the six brightest colonies against antiGFP and anti-V5 antibodies. Colonies No. 2 and No. 3 presented strong bands in both Western blots.

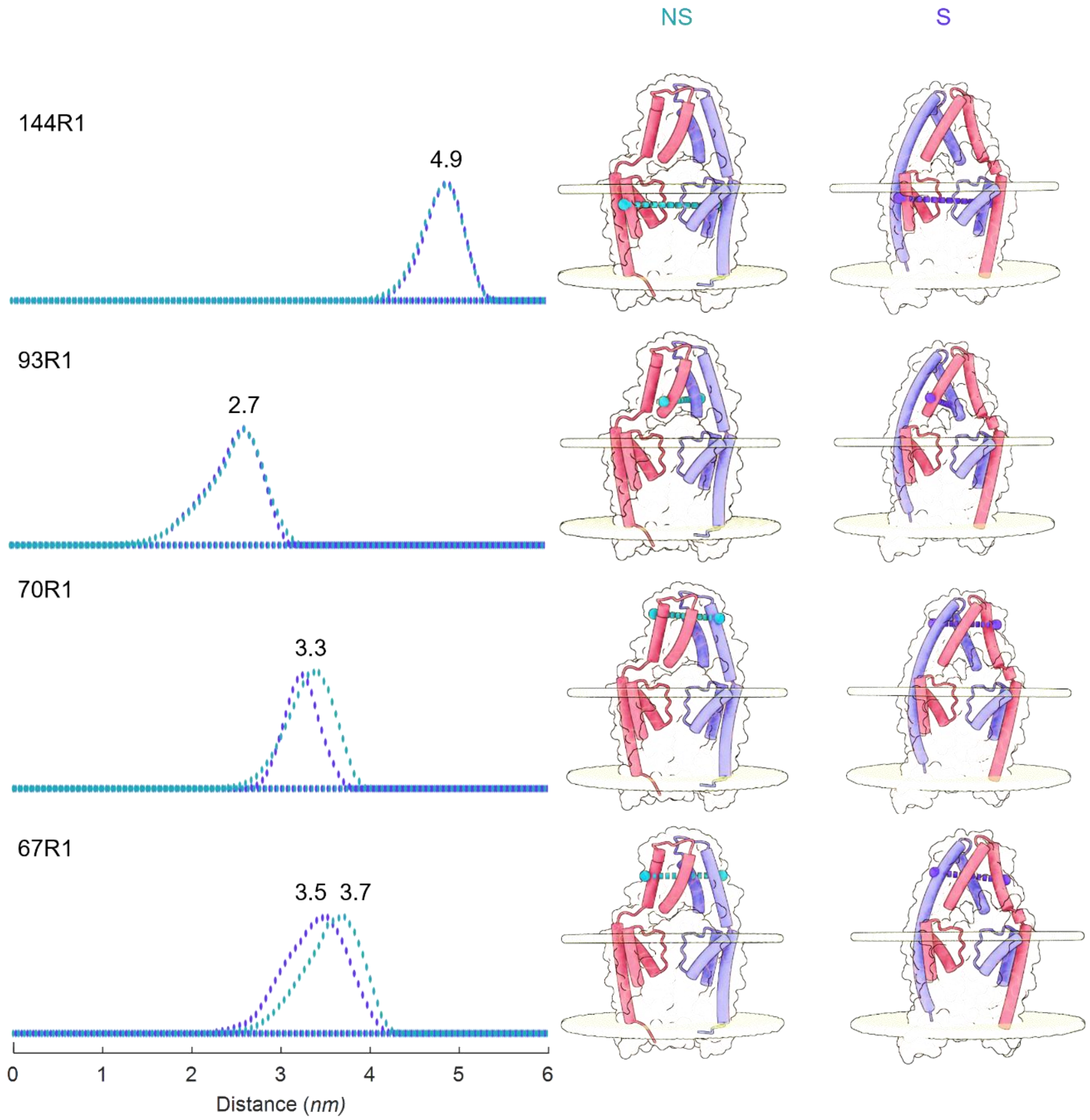

NS
S
144R1
4.9
93R1
2.7
70R1
3.3
67R1
3.5
3.7
0
1
2
3
4
5
6
Distance (nm)

Supplementary Fig. 3: *In silico* distance distributions for spin labelled pairs of homo-dimeric TRAAK. The distance distributions of each labelling pair under the NS state (cyan) and the S state (purple) are shown on the left, while illustrations of the labelling sites for both states are shown on the right.

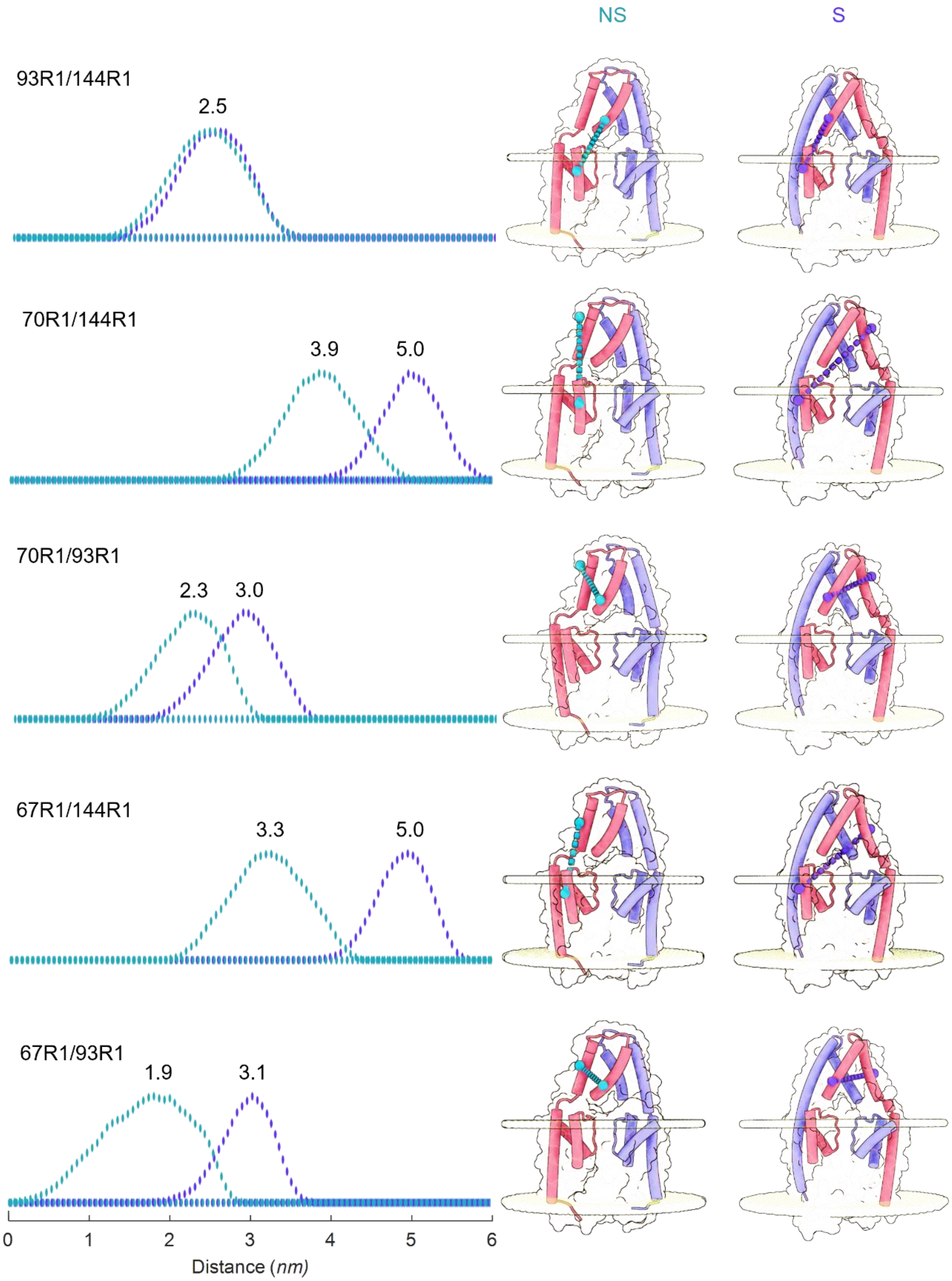
NS
S
93R1/144R1
2.5
70R1/144R1
3.9
5.0
70R1/93R1
2.3
3.0
67R1/144R1
3.3
5.0
67R1/93R1
1.9
3.1
0
1
2
3
4
5
6
Distance (nm)

Supplementary Fig. 4: *In silico* distance distributions for spin labelled pairs of a single subunit of TRAAK. The distance distributions of each labelling pair under the NS state (cyan) and the S state (purple) are shown on the left, while illustrations of the labelling sites for both states are shown on the right.

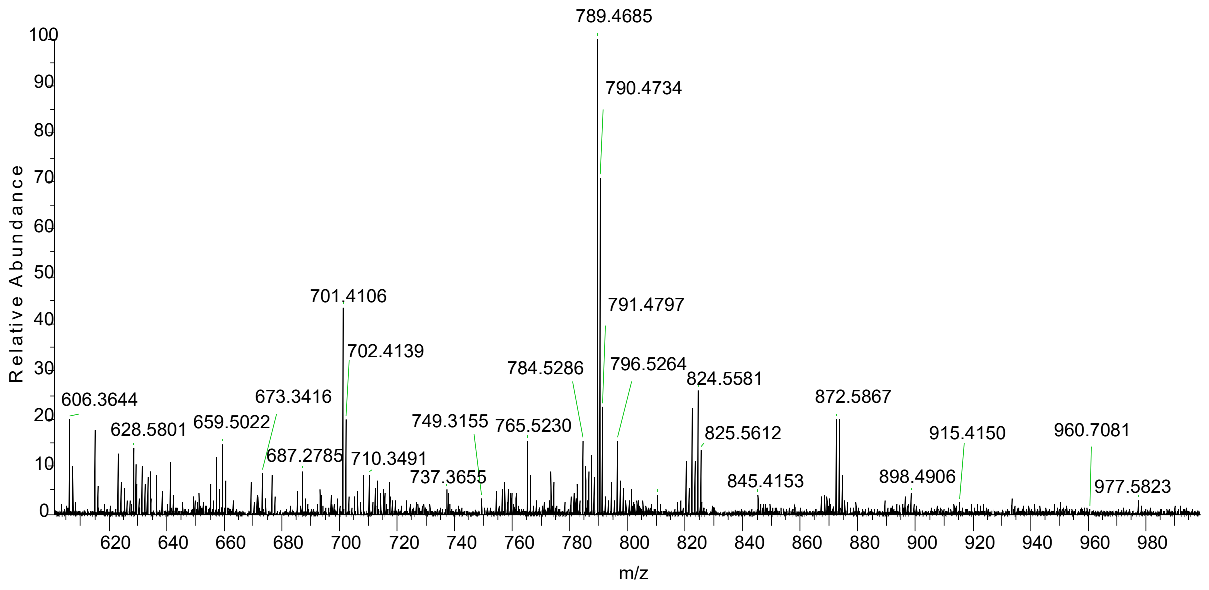

789.4685
790.4734
791.4797
701.4106
702.4139
784.5286
796.5264
824.5581
825.5612
606.3644
673.3416
628.5801
659.5022
687.2785
710.3491
749.3155
765.5230
737.3655
845.4153
872.5867
898.4906
915.4150
960.7081
977.5823
Relative Abundance
m/z

Supplementary Fig. 5: Lipidomics analysis of parental plasma cell membranes of SMALPs-encapsulated TRAAK. Positive survey scan (600-1000m/z).

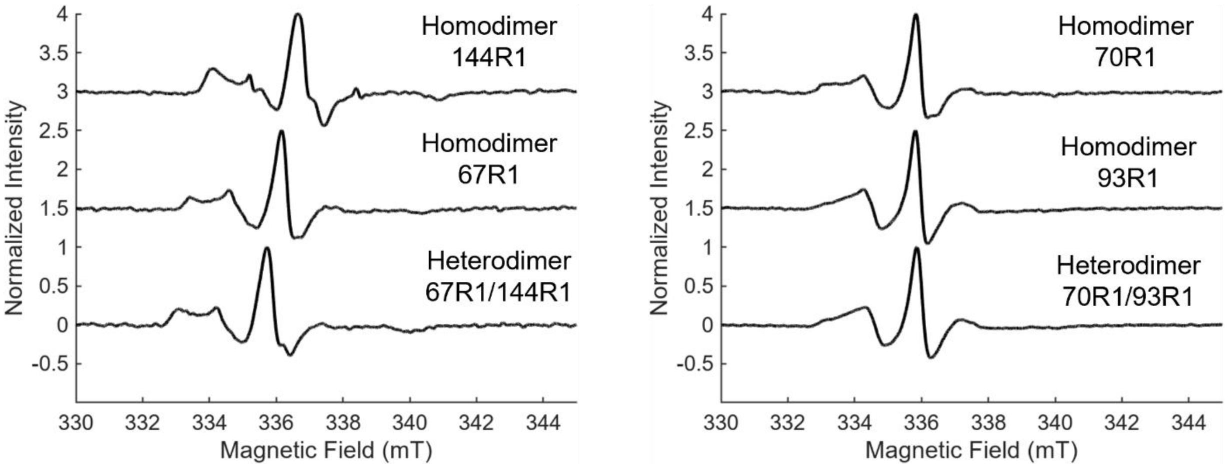
Homodimer
144R1
Homodimer
67R1
Heterodimer
67R1/144R1
Normalized Intensity
Magnetic Field (mT)
Homodimer
70R1
Homodimer
93R1
Heterodimer
70R1/93R1
Normalized Intensity
Magnetic Field (mT)

Supplementary Fig. 6: CW-EPR spectra of spin-labelled TRAAK homodimers and heterodimers in SMALPs. Continuous-wave (CW) EPR spectra recorded at 4 °C for various spin-labelled TRAAK constructs incorporated into SMALPs. Spectra correspond to the labelling sites indicated in the figure. R1 denotes the nitroxide side chain formed by the reaction of MTSSL with engineered cysteine residues.

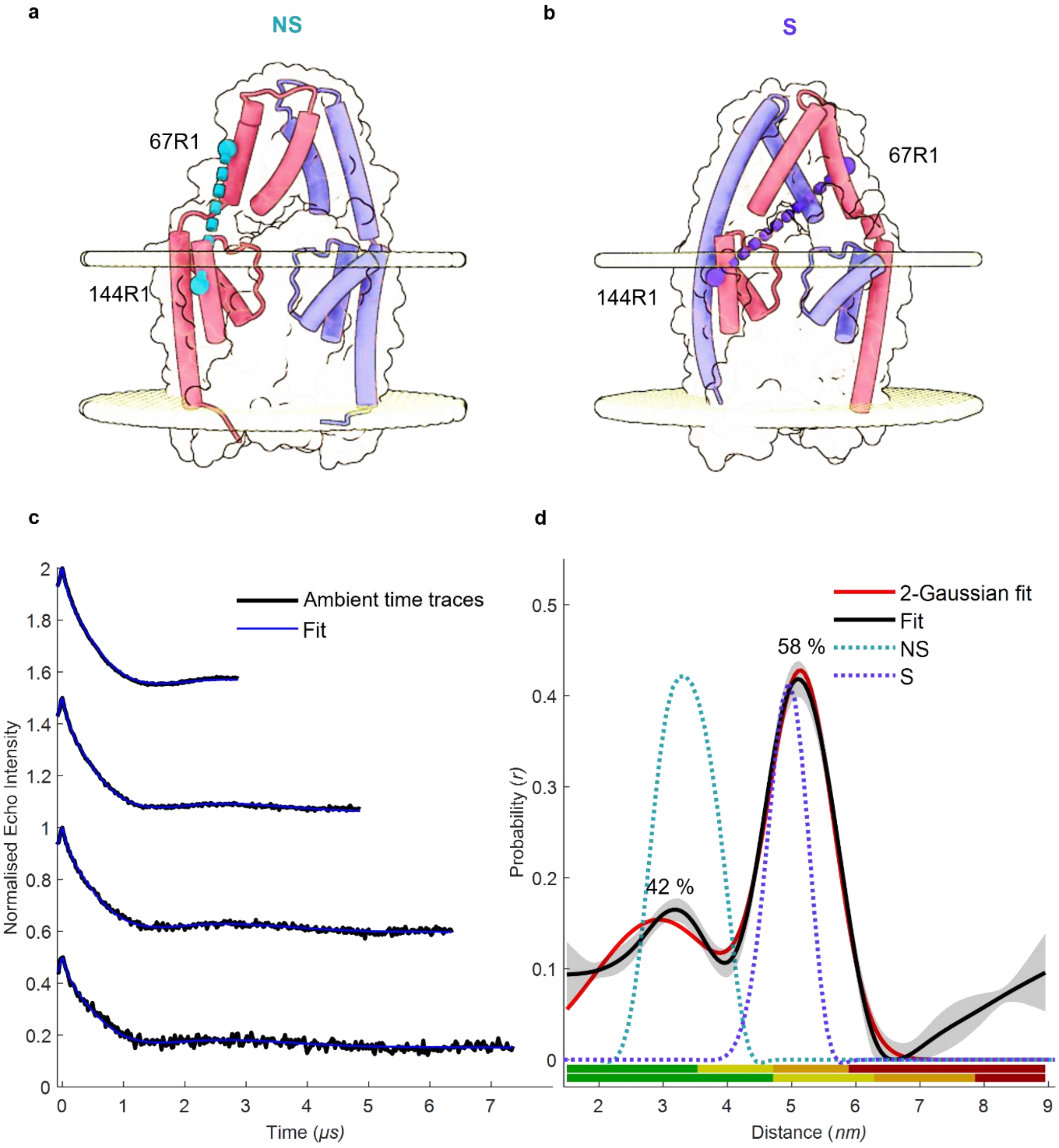

a
NS
67R1
144R1
b
S
67R1
144R1
c
Ambient time traces
Fit
Normalised Echo Intensity
Time (μs)
d
2-Gaussian fit
Fit
NS
S
58 %
42 %
Probability (r)
Distance (nm)

Supplementary Fig. 7: Monitoring the complete TRAAK cap conformational ensemble at ambient temperature by PDS and HSS-SL. a, Illustration of the position of hetero spin labelled pair 67R1/144R1 located on one TRAAK subunit in the NS and S conformations (**b**). **c**, Raw (background uncorrected) PDS time-domain traces (black) with fit (blue) of heterodimeric TRAAK 67R1/144R1 in SMALPs measured at different dipolar evolution times. **d**, The distance distribution was analysed by global fitting in DeerLab (80) from the raw data (black) in **c**. The distribution of two populations was fitted by a 2-Gaussian fit in DeerLab (red) and respective errors were calculated accordingly, with shaded areas corresponding to the 95% confidence interval (see Methods). The *in silico* distance distributions of 67R1/144R1 for the two states are shown as dashed line. The rainbow colour bars represent the reliability of the measured distance ranges (green, shape reliable; yellow, mean and width reliable; orange, mean reliable; red, no quantification possible), the short and long colour bars corresponding to the shortest and longest experimental time window used in **c**. High quality traces allow monitoring the entire TRAAK cap's ensemble, concluding that both S and NS cap states exist in membranes with the S state being the dominant species at ambient temperature (*i.e.* 58/42).

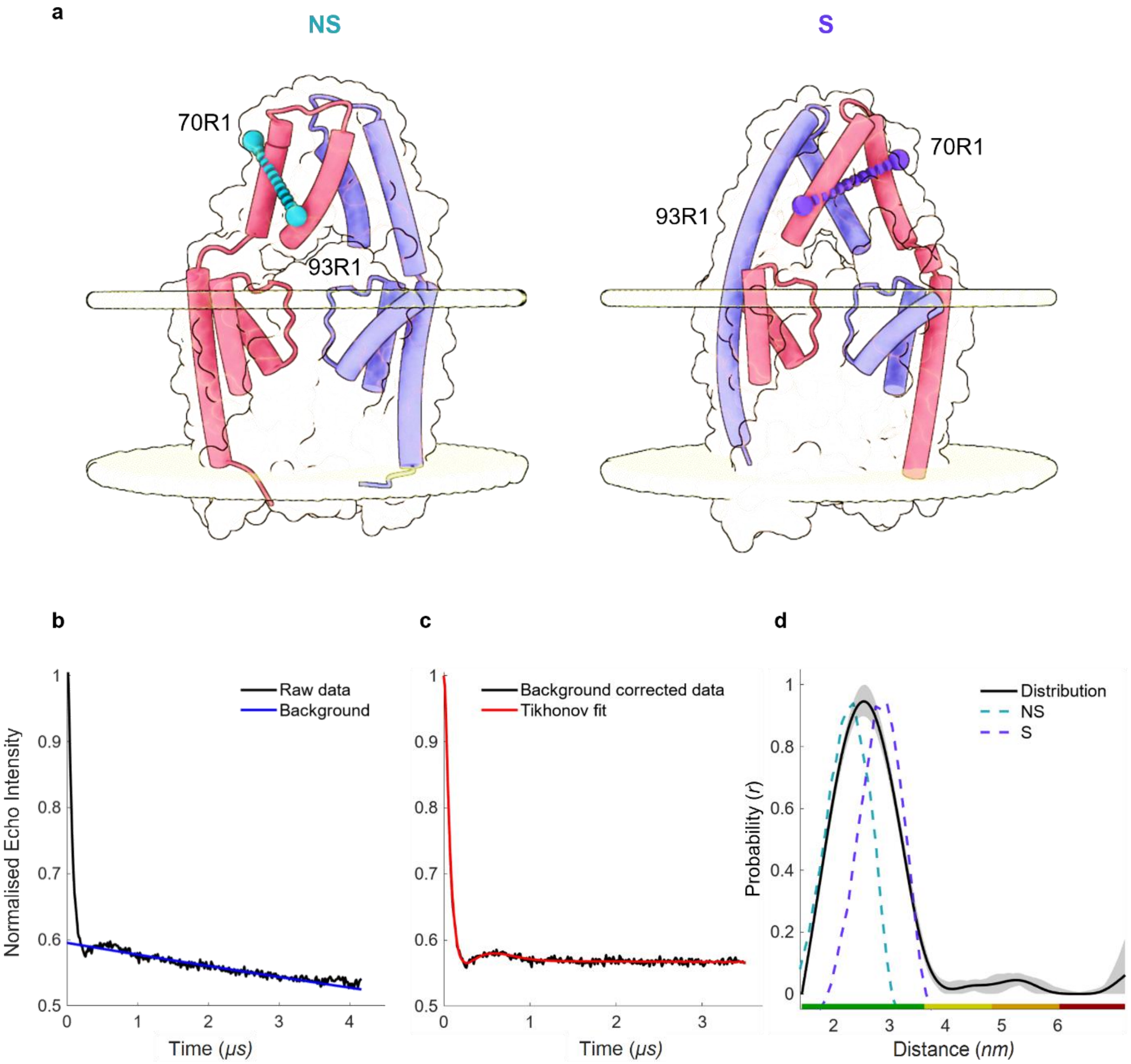

a
NS
S
70R1
93R1
70R1
93R1
b
c
d
Raw data
Background
Normalised Echo Intensity
Time (μs)
Background corrected data
Tikhonov fit
Time (μs)
Distribution
NS
S
Probability (r)
Distance (nm)

Supplementary Fig. 8: PDS distance measurements on the heterodimeric (heterologous single subunit labelling) TRAAK 70R1/93R1 pair. a, Positions of 70R1 and 93R1 on TRAAK under NS and S states. **b**, PDS traces (black) fitted with the corresponding background decay (blue). **c**, PDS traces after background correction (black), along with fits obtained through Tikhonov regularisation (red). **d**, Distance distributions (black) and *in silico* distance distributions under NS (cyan dashed line) and S (purple dashed line) states. Gray shaded areas represent the mean ± 2σ confidence intervals of the measured distributions (calculated using the DeerAnalysis validation tool). The rainbow-colored bars indicate the reliability of the measured distance ranges (green: shape reliable; yellow: mean and width reliable; orange: mean reliable; red: no quantification possible).

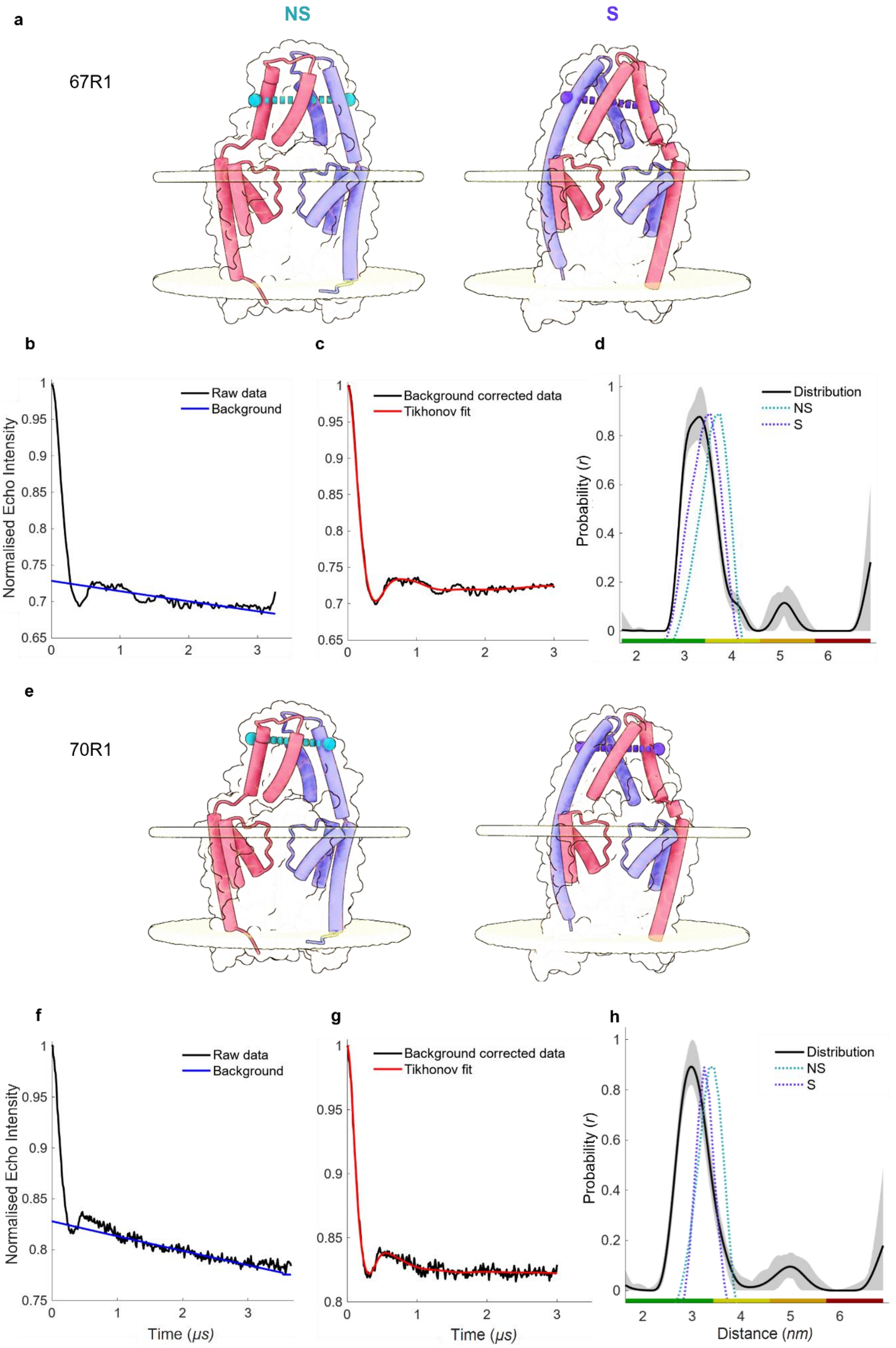

a
NS
S
67R1
b
c
d
Raw data
Background
Normalised Echo Intensity
Background corrected data
Tikhonov fit
Distribution
NS
S
Probability (r)
e
70R1
f
g
h
Raw data
Background
Normalised Echo Intensity
Time (μs)
Background corrected data
Tikhonov fit
Time (μs)
Distribution
NS
S
Probability (r)
Distance (nm)

Supplementary Fig. 9: PDS distance measurements on homo-dimeric TRAAK single pairs 67R1 and 70R1. **a**, **e**, Positions of 67R1 and 70R1 on TRAAK under NS and S states. **b**, **f**, PDS traces (black) fitted with the corresponding background decay (blue). **c**, **g**, PDS traces after background correction (black), along with fits obtained through Tikhonov regularisation (red). **d**, **h**, Distance distributions (black) and *in silico* distance distributions under NS (cyan dashed line) and S (purple dashed line) states. Gray shaded areas represent the mean ± 2σ confidence intervals of the measured distributions (calculated using the DeerAnalysis validation tool). The rainbow-colored bars indicate the reliability of the measured distance ranges (green: shape reliable; yellow: mean and width reliable; orange: mean reliable; red: no quantification possible).

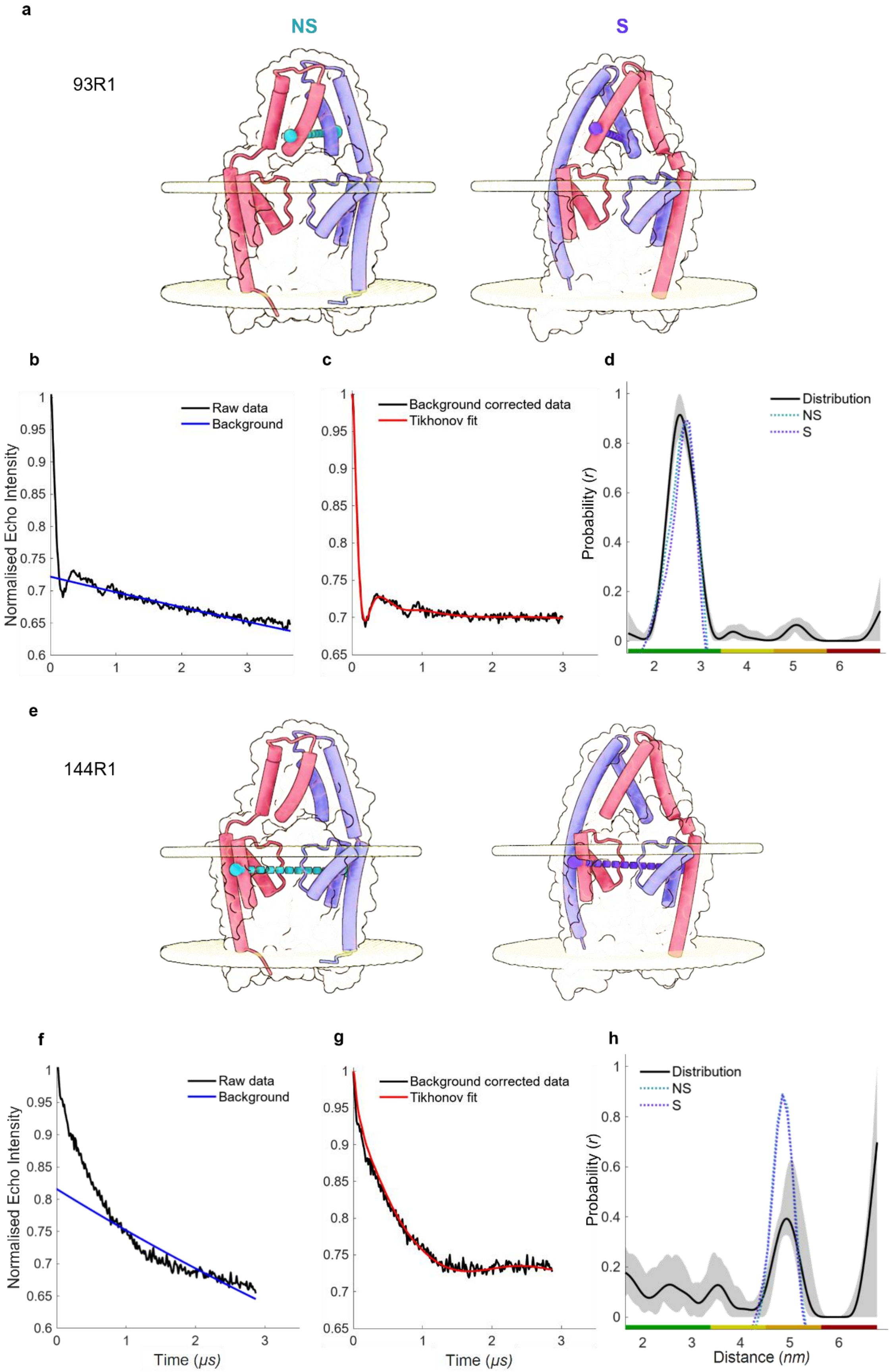

a
NS
S
93R1
b
Normalised Echo Intensity
Raw data
Background
c
Background corrected data
Tikhonov fit
d
Probability (r)
Distribution
NS
S
e
144R1
f
Normalised Echo Intensity
Raw data
Background
Time (μs)
g
Background corrected data
Tikhonov fit
Time (μs)
h
Probability (r)
Distribution
NS
S
Distance (nm)

Supplementary Fig. 10: PDS distance measurements on homo-dimeric TRAAK single pairs 93R1 and 144R1. **a**, **e**, Locations of 93R1 and 144R1 TRAAK sites in the NS and S states. **b**, **f**, PDS traces (black) fitted with the corresponding background decay (blue). **c**, **g**, PDS traces after background correction (black), along with fits obtained through Tikhonov regularisation (red). **d**, **h**, Distance distributions (black) and *in silico* distance distributions under NS (cyan dashed line) and S (purple dashed line) states. Gray shaded areas represent the mean ± 2σ confidence intervals of the measured distributions (calculated using the DeerAnalysis validation tool). The rainbow-colored bars indicate the reliability of the measured distance ranges (green: shape reliable; yellow: mean and width reliable; orange: mean reliable; red: no quantification possible).

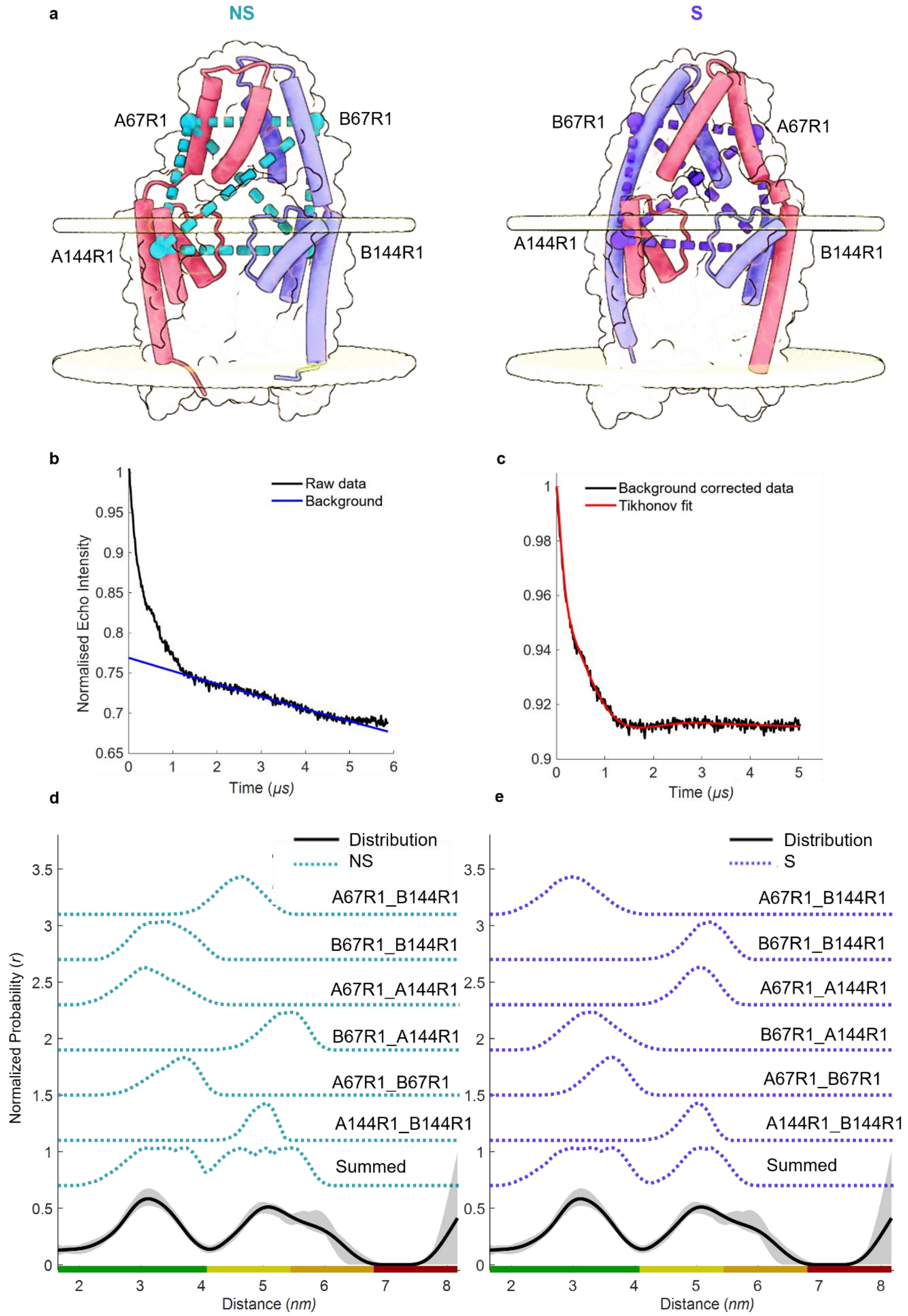
a
NS
S
A67R1
B67R1
A144R1
B144R1
b
Raw data
Background
Normalised Echo Intensity
Time (μs)
c
Background corrected data
Tikhonov fit
Time (μs)
d
Distribution
NS
A67R1_B144R1
B67R1_B144R1
A67R1_A144R1
B67R1_A144R1
A67R1_B67R1
A144R1_B144R1
Summed
Normalized Probability (r)
Distance (nm)
e
Distribution
S
A67R1_B144R1
B67R1_B144R1
A67R1_A144R1
B67R1_A144R1
A67R1_B67R1
A144R1_B144R1
Summed
Distance (nm)

Supplementary Fig. 11: PDS distance measurements on the homodimeric (double subunit labelling) TRAAK 67R1/144R1 pair. a, Positions of 67R1 and 144R1 on TRAAK under NS and S states. **b**, PDS trace (black) fitted with the corresponding background decay (blue). **c**, PDS traces after background correction (black), along with fits obtained through Tikhonov regularisation (red). **d**, and **e**, Measured distance distributions (black) and *in silico* distance distributions under NS (cyan dashed lines) and S (purple dashed lines) states. Under each state, six distance distributions (stacked dashed lines) are generated from four labelling sites, and the averaged distribution, containing all six distance distributions, aligns well with the measured distance distribution. Gray shaded areas represent the mean ± 2σ confidence intervals of the measured distributions (calculated using the DeerAnalysis validation tool). The rainbow-colored bars indicate the reliability of the measured distance ranges (green: shape reliable; yellow: mean and width reliable; orange: mean reliable; red: no quantification possible).

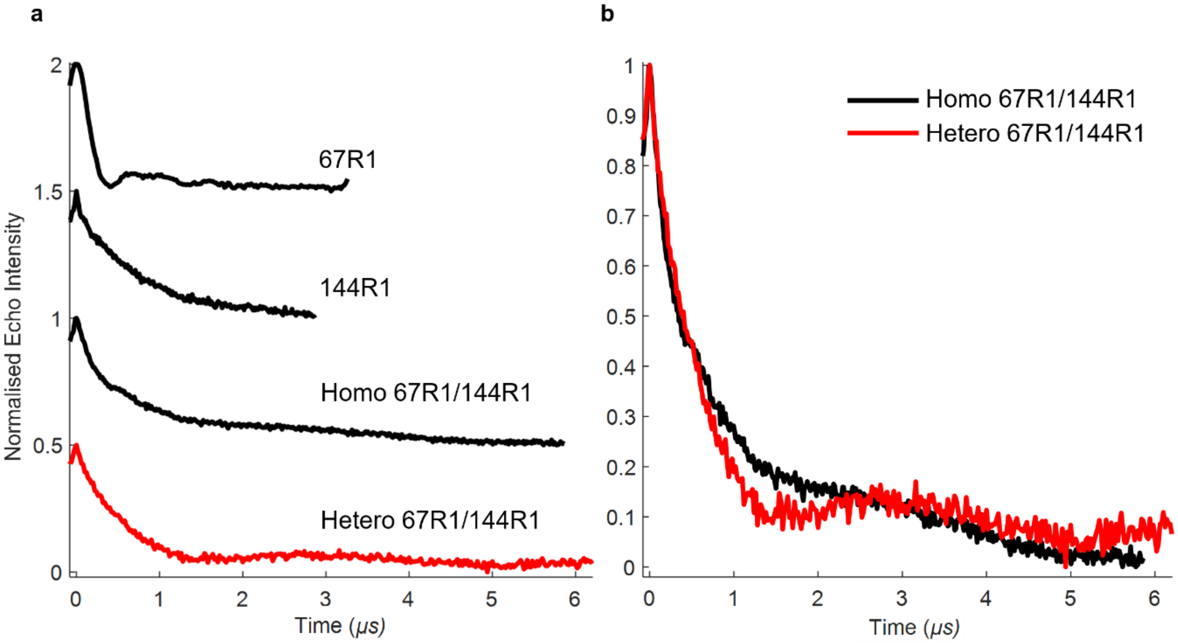

a
b
Normalised Echo Intensity
67R1
144R1
Homo 67R1/144R1
Hetero 67R1/144R1
Time (μs)
Time (μs)
Homo 67R1/144R1
Hetero 67R1/144R1

Supplementary Fig. 12: Comparison of the PDS raw traces for homo double-subunit 67R1, 144R1, 67R1/144R1 and hetero single-subunit 67R1/144R1 spin labelled mutants. **a**, Raw PDS data of TRAAK recorded with different labelling sites from single-labelling pairs 67R1, 144R1, homo 67R1/144R1, and hetero 67R1/144R1 (red). The data are normalized and vertically offset for better visualisation. **b**, Comparison of the normalized raw data of homo 67R1/144R1 and hetero 67R1/144R1. The raw data show that hetero 67R1/144R1 is distinct from each of the others.

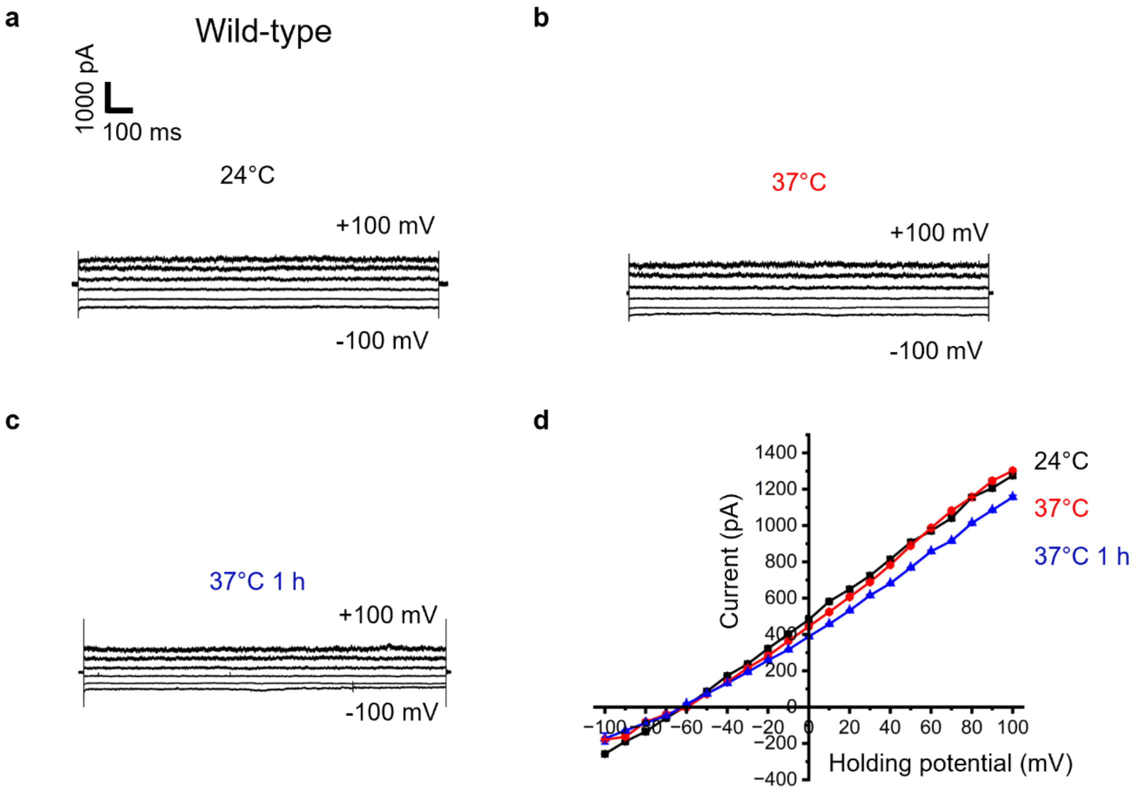

a
Wild-type
1000 pA
100 ms
24°C
+100 mV
-100 mV
b
37°C
+100 mV
-100 mV
c
37°C 1 h
+100 mV
-100 mV
d
Current (pA)
Holding potential (mV)
24°C
37°C
37°C 1 h

Supplementary Fig. 13: TRAAK is not activated by heat in an inside-out patch configuration. **a,** Currents from an excised inside-out patch of wild-type TRAAK recorded with a voltage-step protocol at ambient temperature (24 °C; Vh=0 mV; −100 to +100 mV;ΔV=10 mV; displayed every 40 mV). **b**, **c,** Recordings from the same patch after the bath was raised to 37 °C (~15 min) and after 1 h at 37 °C, respectively. **d**, I–V relationships from the same patch under the three conditions.

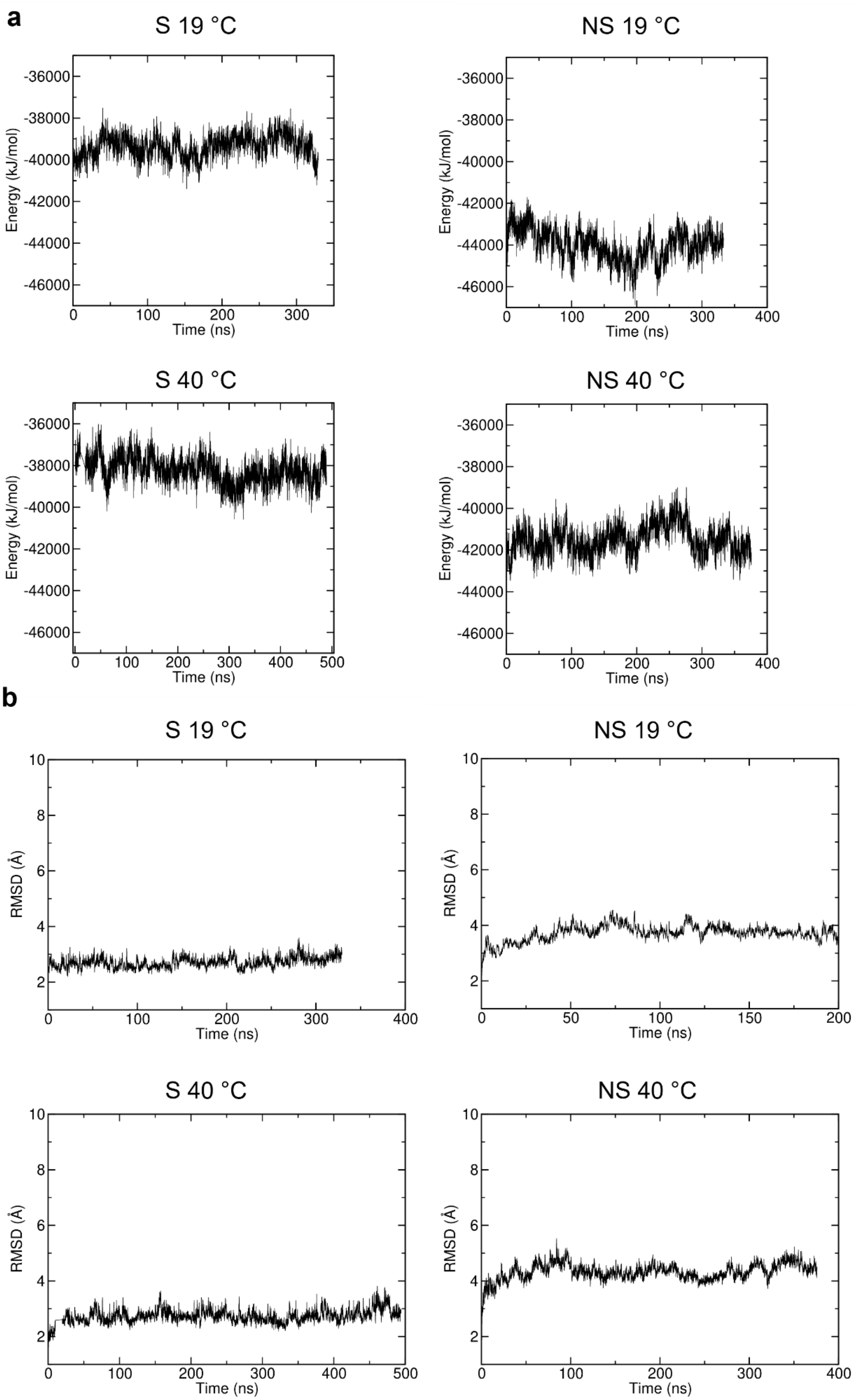
a
S 19 °C
NS 19 °C
S 40 °C
NS 40 °C
Energy (kJ/mol)
-36000
-38000
-40000
-42000
-44000
-46000
Time (ns)
0
100
200
300
400
500
b
S 19 °C
NS 19 °C
S 40 °C
NS 40 °C
RMSD (Å)
10
8
6
4
2
Time (ns)
0
50
100
150
200
300
400
500

Supplementary Fig. 14: Molecular dynamics energy calculations of the S and NS TRAAK states at two different temperatures. Time traces of (**a**) nonbonded interaction energies (Lennard–Jones and Coulomb electrostatic contributions) and (**b**) backbone RMSD between membrane-embedded TRAAK and its environment, compared across temperatures and channel states.

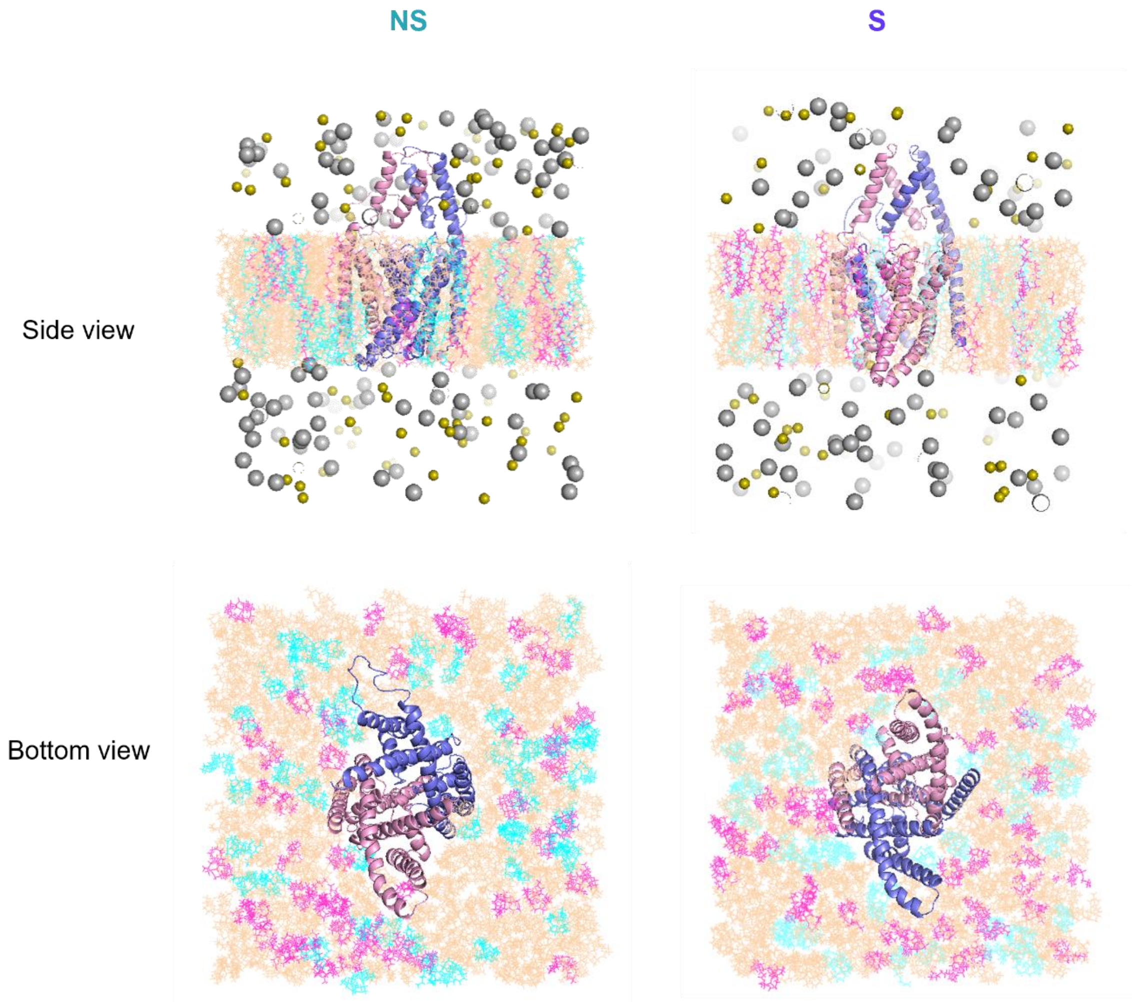

NS
S
Side view
Bottom view

Supplementary Fig. 15: Representative snapshots showing NS and S TRAAK within the bilayer of the simulation box. Water molecules are not shown for clarity. Grey spheres represent K^+ ions, and olive-green spheres represent Cl^- ions. DOPC lipids are shown in wheat, POPS lipids in pink, and POPE lipids in cyan.

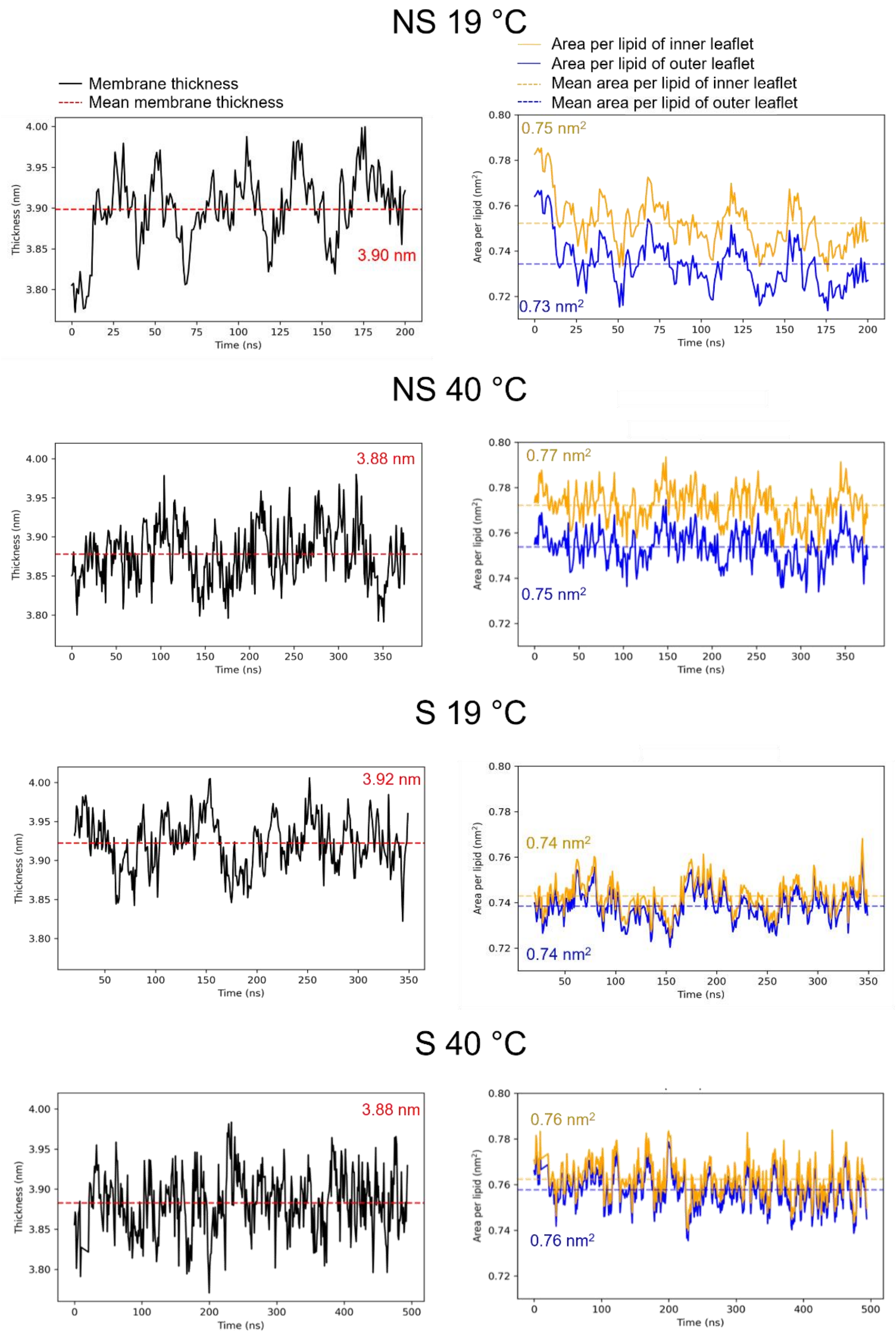
NS 19 °C
Membrane thickness
Mean membrane thickness
Area per lipid of inner leaflet
Area per lipid of outer leaflet
Mean area per lipid of inner leaflet
Mean area per lipid of outer leaflet
3.90 nm
0.75 nm²
0.73 nm²
NS 40 °C
3.88 nm
0.77 nm²
0.75 nm²
S 19 °C
3.92 nm
0.74 nm²
0.74 nm²
S 40 °C
3.88 nm
0.76 nm²
0.76 nm²
Thickness (nm)
Area per lipid (nm²)
Time (ns)

Supplementary Fig. 16: Time evolution of membrane thickness and area per lipid for NS and S TRAAK simulations at different temperatures. In each row, the left panel shows the instantaneous bilayer thickness as a function of time; the red dashed lines and red numbers indicate the time-averaged thickness. The right panels show the corresponding area per lipid for the outer (gold) and inner (blue) leaflet of the bilayer, with horizontal dashed lines and coloured numbers indicating the respective time-averaged values.

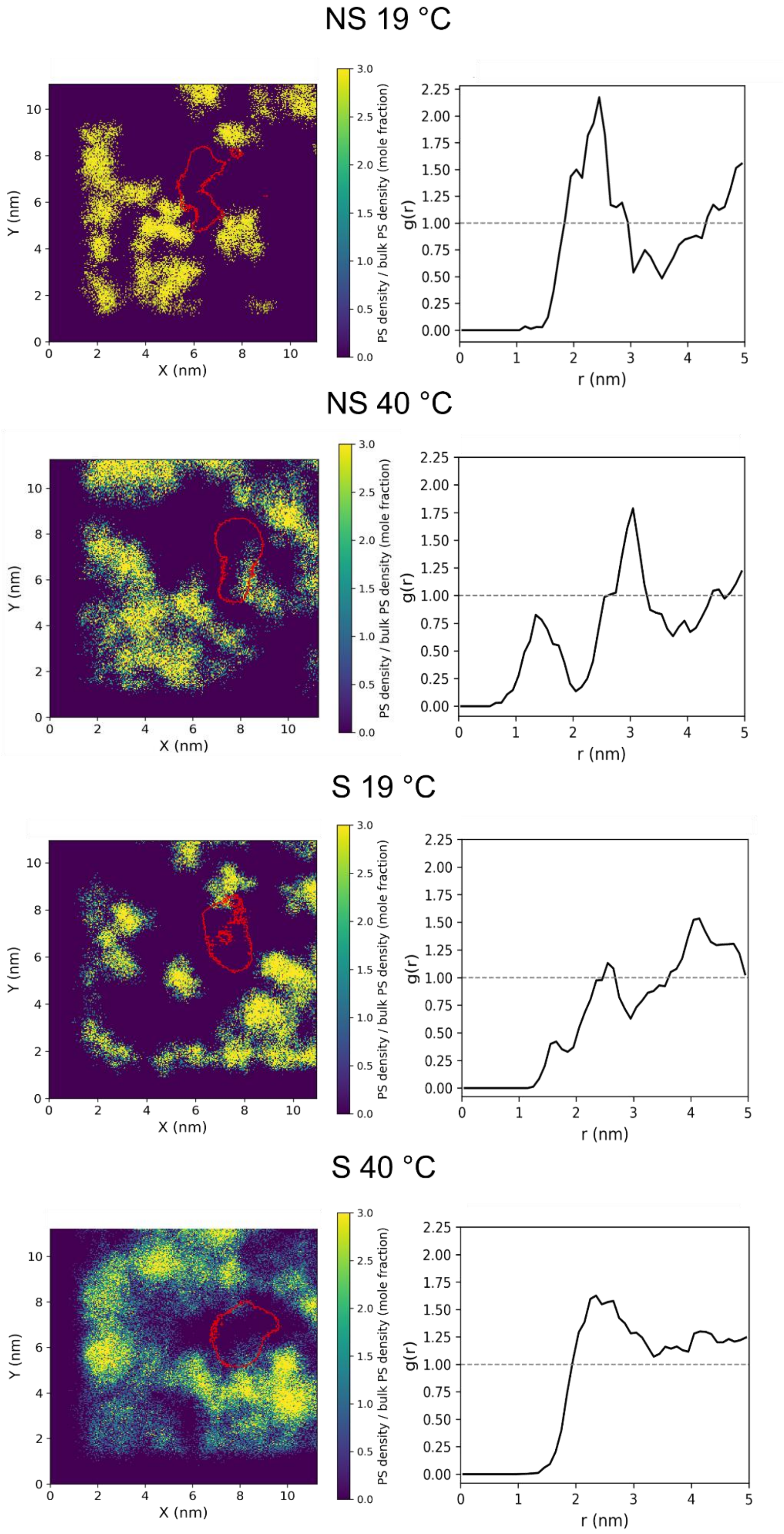
NS 19 °C
NS 40 °C
S 19 °C
S 40 °C
X (nm)
Y (nm)
PS density / bulk PS density (mole fraction)
r (nm)
g(r)

Supplementary Fig. 17: 2D density maps of PS lipid headgroups and radial distribution functions for the inner leaflet of the NS and S TRAAK cap states at different temperatures. The left panels show the local PS headgroup mole fraction in the membrane plane (X–Y), normalised by the bulk PS mole fraction (colour bar; values > 1 indicate enrichment, values < 1 indicate depletion). The red contour indicates the projected outline of the protein complex. The right panels show the corresponding radial distribution function g(r) of PS around the protein, with the dashed line marking g(r) = 1 (bulk density), quantifying PS enrichment or depletion in the inner leaflet.

Supplementary Table 1: K2P channel structures solved to date and their properties. Experimental conditions for obtaining the structures are indicated, including detergents [n-dodecyl-β-D-maltoside (DDM), n-decyl-β-D-maltoside (DM), octyl glucose neopentyl glycol (OGNG), n-heptyl-β-D-thioglucoside (HTG), n-undecyl-β-D-maltopyranoside (UDM)]; lipids [cholesteryl hemisuccinate (CHS), 1,2-dioleoyl-sn-glycero-3-phosphate (DOPA), 1,2-dioleoyl-sn-glycero-3-phosphoethanolamine (DOPE)]; ligands [ML335, norfluoxetine, brominated fluoxetine, BAY 1000493, BAY 2341237]; antigen-binding fragment (Fab); nanobodies [Nb-Binder-58, Nb-Inhibitor-61, Nb-Activator-67, Nb-Activator-76]; and nanodiscs [MSP1D1].

K2P	Organism	Expression system	Method	Cap state	Conditions	Resolution (Å)	PDB (Ref)
**TRAAK**	Homo sapiens	*P. pastoris*	X-ray	Non-swapped	DDM	3.31	3UM7[1]
				Swapped	DDM Fab	2.75	4I9W[29]
					DM Fab	2.50	4WFE[2]
					DM Fab	2.50	4WFF[2]
					DM Fab Ti$^+$	3	4WFG[2]
					DM Fab Ti$^+$	3.01	4WFH[2]
					DDM G124I	3.3	4RUE[30]
					DDM W262S	3.4	4RUF[30]
					DM Fab A270P	2.78	7LJ4[25]
					DM Fab A198E	2.26	7LJ5[25]
					DM Fab Ti$^+$ A198E	2.77	7LJA[25]
					DM Fab G158D	2.97	7LJB[25]
	Mus musculus				DDM Fab	2.77	6PIS[17]
**TREK1**	Homo sapiens	*S. frugiperda*	X-ray	Swapped	-	2.6	4TWK
	Mus musculus	*p. pastoris*			OGNG, HTG, CHS	3.10	6CQ6[34]
					OGNG, HTG, CHS, ML335	3	6CQ8[34]
					OGNG, HTG, CHS	2.8	6CQ9[34]
					OGNG, HTG, CHS I110D	3.40	6V36[35]
					OGNG, HTG, CHS 0 mM K^+	3.88	6W7B[35]
					OGNG, HTG, CHS 1 mM K^+	3.4	6W7C[35]
					OGNG, HTG, CHS 10 mM K^+	3.5	6W7D[35]
					OGNG, HTG, CHS 30 mM K^+	3.29	6W7E[35]
					OGNG, HTG, CHS 50 mM K^+	3.6	6W82[35]
					OGNG, HTG, CHS 100 mM K^+	3.9	6W83[35]
					OGNG, HTG, CHS 200 mM K^+	3.7	6W84[35]
					OGNG, HTG, CHS ML335, 200 mM K^+	3.88	6W85[35]
					OGNG, HTG, CHS ML335, 100 mM K^+	3.3	6W86[35]

					OGNG, HTG, CHS ML335, 50 mM K^+	3.2	6W87[35]
					OGNG, HTG, CHS ML335 complex, 30 mM K^+	3.2	6W88[35]
					OGNG, HTG, CHS ML335, 0 mM K^+	3.4	6W8F[35]
					OGNG, HTG, CHS ML335, 10 mM K^+	3	6W8A[35]
					OGNG, HTG, CHS ML335, 1 mM K^+	2.6	6W8C[35]
	Danio rerio		CryoEM		DDM	3.27	8DE7[31]
					DDM/DOPA	2.82	8DE8[31]
					DDM/DOPE	3.40	8DE9[31]
**TREK2**	Homo sapiens	*S. frugiperda*	X-ray	Swapped	OGNG, CHS	3.20	4BW5[32]
					OGNG, CHS	3.8	4XDJ[32]
					OGNG, CHS, norfluoxetine	3.60	4XDK[32]
					OGNG, CHS, bromated fluoxetine	3.5	4XDL[32]
					OGNG, CHS Nb-Binder-58/	3.59	8QZ1[39]
					OGNG, CHS Nb-Inhibitor-61/	3.5	8QZ2[39]
					OGNG, CHS Nb-Activator-67/	2.4	8QZ3[39]
					OGNG, CHS Nb-Activator-76	3.2	8QZ4[39]
**TASK1**	Homo sapiens	*S. frugiperda*	X-ray	Swapped	DM, CHS	3	6RV2[36]
					DM, CHS, BAY 1000493	2.9	6RV3[36]
					DM, CHS, BAY 2341237	3.1	6RV4[36]
			CryoEM		UDM, CHS	3.1	9G9X[38]
**TASK2**	Mus musculus	*P. pastoris*	CryoEM	Swapped	MSP1D1 pH 6.5	3.45	6WLV[37]
					MSP1D1 pH 8.5	3.52	6WM0[37]
**TASK3**	Homo sapiens	*S. frugiperda*	CryoEM	Swapped	DM, CHS	3.3	9G9V[38]
					DM, CHS G239R	2.5	9G9W[38]
**TWIK1**	Homo sapiens	*P. pastoris*	X-ray	Non-swapped	DM	3.40	3UKM[28]
	Rattus norvegicus		CryoEM	Swapped	MSP1D1 pH7.4	3.33	7SK0[33]
					MSP1D1 pH5.5	3.43	7SK1[33]

Supplementary Table 2: Forward (F) and reverse (R) primers for each mutant used in this study.

Primers	Sequence
PmeI linearisation	F 5’ GCT GTC TTG GAA CCT AAT ATG 3’
	R 5’ TGT CAG TTT TGG GCC ATT TG 3’
E67C	F 5’ TGTCTTGGCGAAGTGAGAGAAAAATTCCTGCGTGCTCACCCTTGCG 3’
	R 5’ TTCGCCAAGACAACGCTGTGCTTGCTGTTCATGGGGTTGCTC 3’
E70C	F 5’ TACAAATTCCTGCGTGCTCACCCTTGCG 3’
	R 5’ GCAGGAATTTGCATCTCACTTCGCCAAGTTCACGCTG 3’
D93C	F 5’ TGCGCACTGGGCGGCGGAGCAGATCC 3’
	R 5’ CAGTGCGCATGCTACCTCCTTAATTAGTAAGCCTAATTC 3’
L144C	F 5’TGTTCTTTCATTTTTTACGCACTTGTGGGTATCCCACTGTTTGGCATC 3’
	R 5’ AAAAATAGAGAAACACCTTCCTGCATCAGTCCTTAGGGCCACATTACC 3’
V5 tag fragment	F 5’ GAAAGCCAATCCCAAATCCCC 3’
	R 5’ GGGTTGATAAGCAGGGGAGTCTTG 3’
His tag fragment	F 5’ ACCACCACCACCACTCGAGCATG 3’
	R 5’ GGGTTGATAAGCAGGGGAGTCTTG 3’

Supplementary Table 3: System composition and simulation box dimensions for the four TRAAK conditions. Lipid composition is the molar ratio DOPC:DOPE:POPS (72:14:14). The total number of lipids in both leaflets is indicated. Dimensions are box lengths (X, Y, Z) in nm at the start of production. Temperatures are 40 °C and 19 °C. Simulation lengths are reported in ns.

Structure	Lipid composition (DOPC:DOPE:POPS)	Total number of Lipids	Dimensions (X,Y,Z) (nm)	Temperature (°C)	Simulation length (ns)
3UM7	72:14:14	Upperleaflet:168	11.25,11.25,12.44	40	376
		Lowerleaflet:164	11.09,11.09,12.58	19	339
4WFF	72:14:14	Upperleaflet:165	11.18,11.18,12.70	40	498
		Lowerleaflet:164	11.03,11.03, 12.81	19	349

Supplementary Table 4: Membrane–protein binding interaction energies for the S and NS states at two distinct temperatures. For each state, the energy difference between temperatures is also reported.

States	Temperature (°C)	Energy (kcal mol^{-1})	Energy difference (kcal mol^{-1})
S	19	-37821 ± 500.2	-979 ± 724
	40	-36842 ± 524.3	
NS	19	-42505.2 ± 548.9	-2689 ± 819
	40	-39816.2 ± 609.1	
