# Supplementary material for: Swapped and non-swapped TRAAK states co-exist in membranes at a ratio influenced by temperature
Source: Nat Commun. 2026 Mar 5;17:3522. doi: 10.1038/s41467-026-70027-9 (PMC13087257; doi:10.1038/s41467-026-70027-9)
Supplement: Supplementary file 3 — Reporting Summary [file 41467_2026_70027_MOESM3_ESM.pdf]

nature portfolio

Corresponding author(s): Christos Pliotas

Last updated by author(s): Jan 23, 2026

Reporting Summary

Nature Portfolio wishes to improve the reproducibility of the work that we publish. This form provides structure for consistency and transparency in reporting. For further information on Nature Portfolio policies, see our Editorial Policies and the Editorial Policy Checklist.

Statistics

For all statistical analyses, confirm that the following items are present in the figure legend, table legend, main text, or Methods section.

n/a	Confirmed	
☐	☒	The exact sample size (n) for each experimental group/condition, given as a discrete number and unit of measurement
☐	☒	A statement on whether measurements were taken from distinct samples or whether the same sample was measured repeatedly
☒	☐	The statistical test(s) used AND whether they are one- or two-sided<br>*Only common tests should be described solely by name; describe more complex techniques in the Methods section.*
☒	☐	A description of all covariates tested
☒	☐	A description of any assumptions or corrections, such as tests of normality and adjustment for multiple comparisons
☐	☒	A full description of the statistical parameters including central tendency (e.g. means) or other basic estimates (e.g. regression coefficient) AND variation (e.g. standard deviation) or associated estimates of uncertainty (e.g. confidence intervals)
☒	☐	For null hypothesis testing, the test statistic (e.g. F, t, r) with confidence intervals, effect sizes, degrees of freedom and P value noted<br>*Give P values as exact values whenever suitable.*
☒	☐	For Bayesian analysis, information on the choice of priors and Markov chain Monte Carlo settings
☒	☐	For hierarchical and complex designs, identification of the appropriate level for tests and full reporting of outcomes
☒	☐	Estimates of effect sizes (e.g. Cohen's d, Pearson's r), indicating how they were calculated

Our web collection on statistics for biologists contains articles on many of the points above.

Software and code

Policy information about availability of computer code

Data collection	Bruker Xepr v 2.8 was used for PDS data collection. Clampex 10.7 was used for electrophysiological data collection. CHARMM-GUI's Membrane Builder tool was used to construct the systems for MD simulations.
Data analysis	PDS: Pymol-embedded MtsslWizard, DeerAnalysis 2022, DEERLab . Electrophysiology: Clampfit 10.7. MD simulations: GROMACS versions 2021.5 and 2024.5.

For manuscripts utilizing custom algorithms or software that are central to the research but not yet described in published literature, software must be made available to editors and reviewers. We strongly encourage code deposition in a community repository (e.g. GitHub). See the Nature Portfolio guidelines for submitting code & software for further information.

Data

Policy information about availability of data

All manuscripts must include a data availability statement. This statement should provide the following information, where applicable:

- Accession codes, unique identifiers, or web links for publicly available datasets
- A description of any restrictions on data availability
- For clinical datasets or third party data, please ensure that the statement adheres to our policy

All data are available in the main text or the supplementary materials.
All raw datasets, including PDS spectroscopy time domain traces, electrophysiology data, MD simulations' data and lipidomics data (ES-MS) that support the findings of this study are available in Figshare. Accession codes are given in the Data availability section of the manuscript.

Research involving human participants, their data, or biological material

Policy information about studies with human participants or human data. See also policy information about sex, gender (identity/presentation), and sexual orientation and race, ethnicity and racism.

Reporting on sex and gender	N/a
Reporting on race, ethnicity, or other socially relevant groupings	N/a
Population characteristics	N/a
Recruitment	N/a
Ethics oversight	N/a

Note that full information on the approval of the study protocol must also be provided in the manuscript.

Field-specific reporting

Please select the one below that is the best fit for your research. If you are not sure, read the appropriate sections before making your selection.

☒ Life sciences ☐ Behavioural & social sciences ☐ Ecological, evolutionary & environmental sciences

For a reference copy of the document with all sections, see nature.com/documents/nr-reporting-summary-flat.pdf

Life sciences study design

All studies must disclose on these points even when the disclosure is negative.

Sample size	For PDS measurements, two hetero-mutant pairs were used to support the key findings of the study, and five additional single and double mutant pairs were also measured to control for these findings.<br><br>Sample size of the electrophysiological data is indicated in the manuscript.
Data exclusions	All data was included in the analysis.
Replication	For PDS measurements, as is common practice and recommended by a recent expert guideline, multiple PDS distances were measured on multiple different double mutants of the same protein and interpreted collectively to monitor the protein's conformation in the same measurement environment (Schiemann et al., 2021, JACS). However, to further validate our results, for the main hetero-mutant pair 67R1/144R1, we conducted three biological repeats, with the two most representative ones included in the main and SI material of the manuscript, as well as ten technical repeats among different conditions reported here.
Randomization	Allocation was not random because it was not relevant to this study. Experimental groups were defined by predetermined conditions (e.g., construct, lipid composition, and temperature/ligand).
Blinding	Blinding was not implemented because it was not relevant to this study. The experimental conditions (construct, lipid composition, temperature/ligand) had to be known during data collection, and analyses (EPR, lipidomics and electrophysiology) were based on predefined quantitative criteria with minimal subjective interpretation.

Reporting for specific materials, systems and methods

We require information from authors about some types of materials, experimental systems and methods used in many studies. Here, indicate whether each material, system or method listed is relevant to your study. If you are not sure if a list item applies to your research, read the appropriate section before selecting a response.

Materials & experimental systems

n/a	Involved in the study	
	☒	Antibodies
	☒	Eukaryotic cell lines
☒		Palaeontology and archaeology
☒		Animals and other organisms
☒		Clinical data
☒		Dual use research of concern
☒		Plants

Methods

n/a	Involved in the study	
☒		ChIP-seq
☒		Flow cytometry
☒		MRI-based neuroimaging

Antibodies

Antibodies used

Anti-GFP Antibody (Antibodies, A283795,Rabbit polyclonal antibody to GFP)
Anti-V5 tag (Antibodies, A269661,Rabbit polyclonal antibody to V5 tag)
Goat anti-rabbit HRP Secondary Antibody (Cambridge Bioscience, AC2114)

Validation

All antibodies were obtained from the indicated commercial companies. The validation data and detailed information are available in the manufacturers' websites.

Eukaryotic cell lines

Policy information about cell lines and Sex and Gender in Research

Cell line source(s)

Pichia pastoris (SMD 1163) (Ref: Brohawn et al., 2012, Science 335, 436-441)

Authentication

N/a

Mycoplasma contamination

N/a

Commonly misidentified lines
(See ICLAC register)

N/a

Plants

Seed stocks

N/a

Novel plant genotypes

N/a

Authentication

N/a
